# Sustainability in Electrophysiology: Reducing the Carbon Footprint of the EP Lab

**DOI:** 10.1016/j.jacadv.2025.102300

**Published:** 2025-10-23

**Authors:** Amro Aglan, Sojin Youn Wass, Sarju Ganatra, Sourbha Dani, Sanjay Rajagopalan, Sadeer Al-Kindi

**Affiliations:** a Department of Cardiology, Westchester Medical Center, New York Medical College, Valhalla, New York, USA; b Department of Cardiovascular Medicine, Cleveland Clinic, Cleveland, Ohio, USA; c Department of Cardiology, Lahey Hospital and Medical Center, Burlington, Massachusetts, USA; d Harrington Heart and Vascular Institute, University Hospitals and School of Medicine, Case Western Reserve University, Cleveland, Ohio, USA; e Center for Health & Nature and Department of Cardiology, Houston Methodist Hospital, Houston, Texas, USA

**Keywords:** climate change, carbon footprint, electrophysiology, health care emissions, sustainability

The World Health Organization has described climate change as the “greatest threat to global health in the 21st century.” Through rising temperatures, air pollution, and extreme weather events, climate change is associated with higher cardiovascular morbidity and mortality, including ischemic heart disease, heart failure, and arrhythmias.^1^ Paradoxically, the health care sector itself accounts for an estimated 5% of global greenhouse gas emissions (up to 10% in developed countries).^2^ Cardiology—one of the most technology-driven specialties—accounts for a substantial share,^3^ with electrophysiology (EP) labs providing a clear example through procedures such as ablation and device implantation that rely heavily on single-use equipment and energy-intensive infrastructure. This environmental impact is expected to grow with the rising volume of EP procedures, a trajectory likely to continue with population aging and advancing arrhythmia therapies. Yet, sustainability data in EP labs remain limited, with few procedure-level analyses or life-cycle assessments available. Recognizing climate change as a threat to cardiovascular health should motivate the cardiology community to address its footprint with urgency. This viewpoint reviews key drivers of carbon emissions in EP labs, highlights practical mitigation strategies, and calls on clinicians, hospitals, societies, and industry to collaborate toward a more sustainable future in cardiac EP.

EP laboratories are among the most resource- and energy-intensive environments in cardiovascular care. They often operate for extended hours and rely on continuous HVAC (Heating, Ventilation, and Air Conditioning) systems to maintain sterility. Advanced imaging equipment such as fluoroscopy, mapping consoles, and intracardiac echocardiography (ICE), along with single-use catheters, cables, and anesthesia systems, contribute to high energy demands and substantial medical waste. This constant demand increases the carbon footprint, particularly when powered by fossil fuels.

Disposable devices and their packaging are major drivers of the environmental footprint of EP. A typical atrial fibrillation (AF) ablation often requires multiple single-use components, including catheters, sheaths, introducers, defibrillator pads, and three-dimensional (3D)-mapping reference patches. Each AF ablation has been estimated to emit approximately 77 kg CO_2_-equivalent, about 75% from disposable materials, with catheters contributing ∼38%.^4^ Packaging is also substantial; for example, 62% of the carbon footprint of a transseptal needle derives from its packaging.^4^ Importantly, device-use practices vary across countries and institutions. While diagnostic and mapping catheters are increasingly reprocessed in many centers, this practice is not universally authorized. Radiofrequency ablation catheters may also be reprocessed in certain settings, whereas pulsed field ablation catheters remain strictly single-use by current labeling and policy. Reflecting this heterogeneity, a recent European survey reported that more than 50% of diagnostic and ablation catheters are discarded after a single use, fewer than 20% are reused, and 69% of associated packaging is not recycled.^5^

A persistent challenge in addressing these impacts is the lack of transparent, procedure-level data on carbon emissions. Device manufacturers rarely disclose material sourcing or composition, which makes estimations of carbon emissions in life-cycle analysis difficult. Hospitals rarely track emissions, and physicians are often unaware of carbon costs of the products. Despite these challenges, there are concrete and attainable strategies that can reduce the environmental impact of EP labs without compromising patient care. Sustainable strategies can be categorized into four domains: 1) the 3 Rs (reduce, reuse, recycle); 2) device innovation; 3) energy efficiency; and 4) procedural optimization (Figure 1).

**Figure 1:**
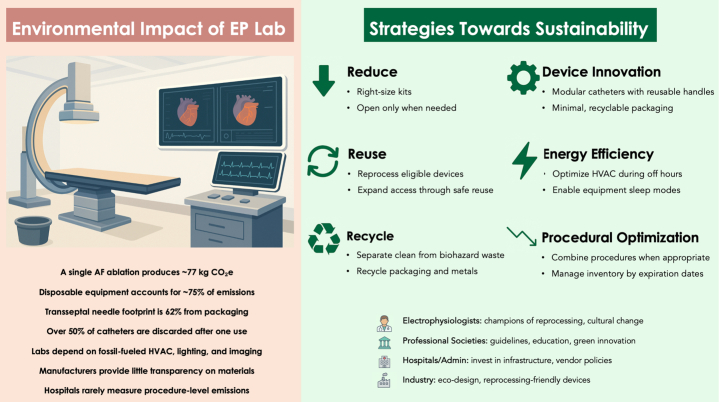
**Environmental Impact of Electrophysiology Labs and Strategies for Sustainability** Major environmental contributors in electrophysiology labs include disposable devices and packaging, with atrial fibrillation ablation emitting ∼77 kg CO_2_-equivalent.^4^ Mitigation strategies include the 3 Rs (reduce, reuse, recycle), device innovation, energy efficiency, and procedural optimization. EP = electrophysiology; AF = atrial fibrillation; HVAC = Heating, Ventilation, and Air Conditioning.

Reducing the consumption of disposable items is one of the simplest ways to decrease emissions. “Right-sizing” procedural kits by removing nonessential items prevent unnecessary waste. Reprocessing of EP equipment offers clear environmental and economic benefits when performed under rigorous oversight. Under both the Food and Drug Administration and European Union Medical Device Regulation frameworks, reprocessors are held to manufacturer-level standards for quality management, validation, traceability, and safety.^6^^,^^7^ Life-cycle analyses show that catheter reprocessing can reduce global-warming impact by ∼50% and raw-material use by ∼30%.^8^ Despite these advantages, adoption remains inconsistent: while many electrophysiologists report experience with reprocessed materials, most diagnostic, mapping, and ablation catheters are still discarded after a single use, with reprocessing permitted in some countries but prohibited in others.^5^ Concerns about sterility, durability, and precision persist, but evidence is reassuring—highlighting the need for rigorous processes and greater physician awareness of safe reprocessing. When implemented correctly, reprocessing has proved safe: ICE catheters have been reused up to 20 times with no infections, saving over 2.25 million euros across ∼1,000 procedures and reducing waste by 95%.^9^ Multinational experience with resterilized pacemakers and defibrillators has shown infection rates comparable to new implants and no device-related deaths.^10^ Beyond cost and environmental gains, reprocessing may promote cardiovascular equity by expanding access to EP therapies in underserved settings. Furthermore, many EP lab materials (such as plastics and cardboard) are recyclable if properly sorted. Separating clean packaging from biohazardous waste and partnering with waste services can greatly reduce landfill and incineration.

Device innovation offers a promising path to more sustainable EP labs by incorporating environmental considerations into product manufacturing and end-of-life management. Examples include modular catheters that allow reuse of handles and cables by making only the distal portion disposable, significantly reducing waste. Streamlined packaging—using right-sized boxes, fewer protective layers, lightweight materials, and digital instructions (eg, QR codes)—further cuts impact. The use of sustainable materials such as biodegradable polymers (eg, plant-based bioplastics), recyclable mono-material plastics such as polyethylene terephthalate or high-density polyethylene, and molded paper trays instead of rigid plastics strengthens these efforts. Looking ahead, innovations such as smart sensors embedded in devices could enable real-time tracking of usage, sterilization, and environmental metrics across the supply chain.

Improving energy efficiency is another opportunity. EP labs can reduce their energy footprint by optimizing HVAC systems. Lowering air exchange rates and temperature settings during off-hours or between cases can save energy while maintaining infection control standards. Modern fluoroscopy systems, mapping consoles, and monitors include standby or sleep modes that can reduce unnecessary power consumption, and these features are increasingly being adopted. Consistent implementation depends on staff training and system automation. Hospitals can further reduce emissions by transitioning to renewable or low-carbon energy sources, thereby lowering the footprint of each procedure.

Another important opportunity lies in procedural optimization and resource management. Clinical pathways should be designed to minimize unnecessary visits, room turnover, and duplicate preparation. Thoughtful procedural planning, including combining procedures when appropriate in selected patients (eg, AF ablation with left atrial appendage closure), may reduce anesthesia exposure, patient travel, and overall resource use. Nonfluoroscopic 3D mapping further decreases fluoroscopy time, radiation, and energy needs. Practical tools such as confirm-before-open checklists and case-specific device menus are already adopted in many centers and can help limit the default opening of unused items.

Improvements in inventory management can further reduce waste. Supplies should be organized and rotated according to expiration dates, ensuring that usable items are not discarded prematurely. In parallel, donation programs provide a meaningful opportunity to extend the life of safe, unused materials. Brand-new, single-use catheters that are inadvertently opened but remain pristine can be donated to resource-limited settings. Formalized donation pathways can reduce landfill burden while expanding access to life-saving therapies, aligning environmental stewardship with broader goals of cardiovascular equity.

Electrophysiologists themselves can lead these changes. In a survey of more than 200 electrophysiologists, 62% expressed a desire to reduce the environmental impact of their work.^5^ With direct control over device use and procedural workflows, EP physicians are well-positioned to lead sustainability efforts. Advocating for reprocessed devices—by requesting, selecting, or approving them when appropriate—is one of the most effective steps they can take. Broader acceptance often depends on leadership from experienced clinicians who trust the evidence supporting the safety and efficacy of reprocessing. Beyond device choices, electrophysiologists can drive institutional change by incorporating environmental metrics into quality dashboards, and leading through example to foster a culture of sustainable practice.

Major cardiovascular professional societies, including the Heart Rhythm Society, American College of Cardiology, American Heart Association, and European Society of Cardiology, can shape clinical standards and culture by issuing guidelines that legitimize sustainability efforts. They can highlight green practices at annual meetings through dedicated sessions, case studies, and research presentations. Workshops on device reprocessing, energy efficiency, and life-cycle assessment can provide practical guidance. Societies can also foster collaboration with industry to promote eco-conscious innovation. Finally, integrating sustainability education into fellowship training ensures the next generation of electrophysiologists adopts environmental stewardship as part of routine practice.

Hospitals and administrators are equally important partners. Yet, surveys identify limited local institutional support as one of the main barriers to adopting sustainable practices.^5^ Administrators can address this by investing in infrastructure such as energy-efficient HVAC systems, light-emitting diode lighting, and effective waste segregation, while also integrating environmental criteria into vendor evaluations to encourage greener products. Although some initiatives may require up-front costs (eg, recycling infrastructure or reprocessing contracts), long-term savings often justify the investment. Tracking the carbon footprint of each procedure—similar to radiation monitoring—would help set measurable targets. Policymakers can accelerate adoption by setting reprocessing standards and rewarding hospitals and clinicians that implement greener practices.

Industry must also be engaged in advancing EP sustainability by innovating products with smaller environmental footprints and openly reporting life-cycle emissions to drive accountability. Sustainability strategies should extend across the product life cycle, from sourcing to disposal or reuse. Key priorities include early approval and support for reprocessing of catheters and cables (particularly for newer products such as four-dimensional ICE catheters) and implementing take-back programs that enable closed-loop recycling of clean packaging waste, with hospitals incentivized to return materials. As environmental impact becomes an increasingly important factor in procurement, companies that prioritize eco-conscious design and transparency will be best positioned to lead.

In summary, EP labs represent the dual challenge and opportunity of health care in the era of climate change. As high-resource, energy-intensive environments, they significantly contribute to the health care carbon footprint. Yet targeted strategies such as reducing disposables, supporting reprocessing, improving energy efficiency, and innovating device design can lower emissions without compromising care. Clinicians, hospitals, societies, and industry all share responsibility. By integrating sustainability into daily practice, EP labs can become a model for climate-conscious cardiology—ensuring the care we deliver protects both our patients and the planet.

## Funding support and author disclosures

The authors have reported that they have no relationships relevant to the contents of this paper to disclose.
